# Characterization of Astaxanthin Nanoemulsions Produced by Intense Fluid Shear through a Self-Throttling Nanometer Range Annular Orifice Valve-Based High-Pressure Homogenizer

**DOI:** 10.3390/molecules26102856

**Published:** 2021-05-12

**Authors:** Gary B. Smejkal, Edmund Y. Ting, Karthik Nambi Arul Nambi, Richard T. Schumacher, Alexander V. Lazarev

**Affiliations:** Pressure BioSciences, South Easton, MA 02375, USA; eting@pressurebiosciences.com (E.Y.T.); KNambi@pressurebiosciences.com (K.N.A.N.); rschumacher@pressurebiosciences.com (R.T.S.); alazarev@pressurebiosciences.com (A.V.L.)

**Keywords:** argan oil, astaxanthin, emulsion, krill oil, nanoemulsion, annular valve, ultra-shear technology

## Abstract

Stable, oil-in-water nanoemulsions containing astaxanthin (AsX) were produced by intense fluid shear forces resulting from pumping a coarse reagent emulsion through a self-throttling annular gap valve at 300 MPa. Compared to crude emulsions prepared by conventional homogenization, a size reduction of over two orders of magnitude was observed for AsX-encapsulated oil droplets following just one pass through the annular valve. In krill oil formulations, the mean hydrodynamic diameter of lipid particles was reduced to 60 nm after only two passes through the valve and reached a minimal size of 24 nm after eight passes. Repeated processing of samples through the valve progressively decreased lipid particle size, with an inflection in the rate of particle size reduction generally observed after 2–4 passes. Krill- and argan oil-based nanoemulsions were produced using an Ultra Shear Technology™ (UST™) approach and characterized in terms of their small particle size, low polydispersity, and stability.

## 1. Introduction

Astaxanthin (AsX) is a natural red pigment synthesized in the algae *Haematococcus pluvialis*. It is responsible for the coquelicot coloration of crustaceans, salmon, and flamingo who feed on these algae. Antarctic krill, the single largest biomass on earth, is a rich source of natural AsX that supplies the food chain. In shellfish, most of the AsX is localized in the shell where it is bound to proteins.

AsX is one of the most powerful antioxidants known to science, with applications in the pharmaceutical, nutraceutical, cosmetic, food, and animal feed industries. The antioxidant activity of AsX is 6000 times higher than that of vitamin C, 800 times higher than that of coenzyme Q, and 100 times higher than that of vitamin E [1,2]. It has been shown to modulate cell signaling pathways involved in cancer [3], to ameliorate oxidative stress [4], and to enhance immune response [5]. AsX can cross the blood–brain barrier and has been shown to increase gene expression of several proteins known to be involved in brain recovery [6]. Recent studies have suggested that AsX may have a positive effect on COVID-19 cytokine storm [7,8]. In cosmetics, AsX repairs UV-damaged skin, but unlike retinoids, it does not produce cytotoxic metabolites.

AsX is practically water insoluble and less than 10% oral bioavailability has been reported [9], undermining its value as a food additive. However, oral bioavailability is significantly improved when astaxanthin is consumed with dietary fats or in lipid-based formulations [9,10]. Thus, the effective use of AsX as a food additive hinges on improving its solubility, stability, and bioaccessibility. For example, the absorption of AsX by Caco-2 cells is significantly increased from AsX-loaded emulsions [11].

Historically, AsX nanoemulsions have been produced by high-pressure homogenization, typically at pressures less than 200 MPa, and characteristically generating particle sizes on the order of 100–200 nm [12,13,14]. In contrast, the ultra shear technology (UST) operates at unprecedented high pressures and flow rates, facilitating the formation of nanoemulsions of small particle size and narrow size distribution. UST has been used to process raw milk at 400 MPa at high flow rates amenable to manufacturing scale processes [15]. This is of particular relevance since there is growing interest in the fortification of dairy and other foods with carotenoids to boost their nutritional value while preserving their organoleptic properties. In addition to improving the solubility and stability of AsX, its encapsulation has also been shown to mask the malodorous taste and smell of natural AsX sourced from krill in yogurt [16].

The aim of this study was to evaluate a rapid and efficient method of preparing stable, optically clarified astaxanthin-containing nanoemulsions using a recently developed high-pressure homogenizer system based on UST.

## 2. Results

### 2.1. Selection of Surfactants

Nanoemulsion particle size distribution and stability are influenced by surfactant type and concentration, and as more numerous and smaller particles are derived, the increase in the surface area requires a higher surfactant concentration to stabilize.

The lecithin phospholipids Phospholipon 85G (PL85G) and Alcolec S were initially investigated as surfactants in nanoemulsion formulations. The hydrophobic–lipophilic balance (HLB) of the lecithin derivatives was adjusted by the addition of a second surfactant of higher HLB, either polyoxyethylene sorbitan monolaurate (PS20) or polyoxyethylene sorbitan monooleate (PS80). PS20 was preferred over PS80 since its higher HLB enabled it to be used at lower concentrations. Final surfactant concentrations were determined empirically from argan oil formulations producing the smallest particle size and highest sample clarity. The total surfactant:oil ratio (SOR) was 0.7 and 0.9 in Alcolec S/PS20 and PL85G/PS20 formulations, respectively.

### 2.2. AsX/Argan Oil Nanoemulsions

Stable, water-miscible AsX nanoemulsions were produced by UST from coarse emulsions, which were cycled through the annular valve. A portion was reserved and analyzed after each cycle. The crude emulsions were prepared by rotor–stator homogenization. AsX was dissolved in the argan oil phase at 1.2 mg/g concentration. The oil phase was 10% AsX/argan oil in the final formulation. Surfactants were 5% PS20 and either 4% PL85G or 4% Alcolec S.

**Figure 1:**
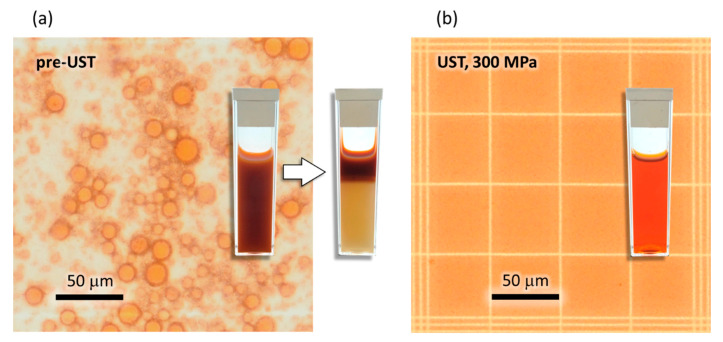
(**a**) Photomicrographs showing AsX/argan oil/PL85G emulsion prepared by rotor-stator homogenization. The mean oil droplet size of the starting material was 5900 ± 3800 nm. Inset shows milky emulsion prepared by rotor–stator homogenization (left) and phase separation that occurs within 48 h (right). (**b**) The same emulsion following UST showing the disappearance of visible oil droplets due to a lipid particle size reduction by nearly two orders of magnitude and below the diffraction limit of visible light. Inset shows stable, clarified nanoemulsion. The bar represents 50 μm.

**Figure 2:**
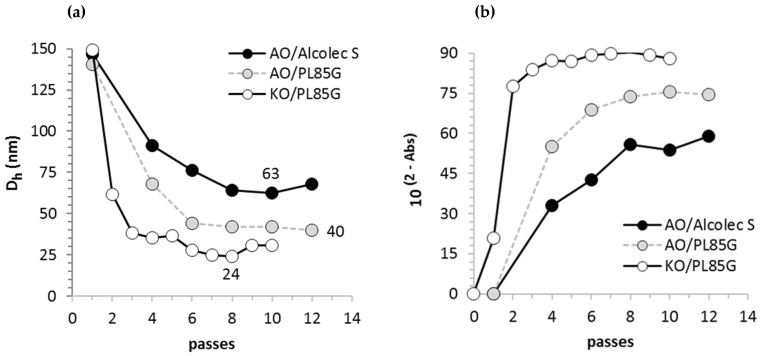
Progressively decreasing particle size commensurate with increased sample clarity of three representative AsX formulations over 1–12 UST passes at 300 MPa. (**a**) Mean hydrodynamic particle diameter (D_h_) of AsX nanoemulsions formed in argan oil (AO) or krill oil (KO) formulations. Surfactants were 5% PS20 and either 4% Alcolec S or 4% PL 85G. The KO/PL85G formulation produced the smallest particle size at the fastest rate (38 nm after three UST cycles) and reached a minimum size of 24 nm after eight cycles. (**b**) Sample clarity quantified by absorbance at 595 nm and expressed as transmittance. KO/PL85G nanoemulsions were most clear due to the smallest particle size obtained in the three formulations.

AsX emulsions prepared by rotor-stator homogenization were milky pink suspensions prone to complete phase separation within 48 h. Particles were too large and polydisperse to be accurately measured by dynamic light scattering (DLS). By optical microscopy, large oil droplets of broad size distribution ranging from 1000–23,000 nm in diameter (5900 ± 3800 nm, median 4800 nm, *n* = 428) were observed in the AsX/argan oil emulsions obtained by the rotor–stator homogenizer method. Following a single pass through the UST valve at 300 MPa, the mean hydrodynamic diameter (D_h_) of particles determined by DLS was decreased to 155 ± 10 nm, confirming a size reduction of nearly two orders of magnitude (Figure 1). In PL85G formulations, particle size progressively decreased from 155 ± 10 nm following a single pass to 51 ± 5 nm after six passes (Figure 2a). A minimum particle size of 40 nm was reached after 12 passes. Compared to PL85G formulations, slightly larger particle sizes were observed in Alcolec S formulations, reaching a minimum size of 60 nm after 10 UST passes.

The transition of the milky pink “smoothie” to stable, optically clear AsX nanoemulsions was marked by decreased Rayleigh light scattering corresponding to a smaller oil droplet size (Figure 2b). Thus, sample clarity measured by UV/Vis spectroscopy at 595 nm was used as an additional metric of nanoemulsification efficiency.

### 2.3. AsX/Krill Oil Nanoemulsions

Krill oil-based formulations yielded the smallest particle size of the three formulations investigated. AsX concentration in krill oil was 0.4 mg/g. The oil phase was 10% krill oil in the final formulation. Surfactants were 5% PS20 and 4% PL 85G. 

Large oil droplets formed in crude AsX/krill oil emulsions prepared by conventional homogenization had an estimated diameter of 17,300 ± 7300 nm (median = 16,300 nm, *n* = 274) which was reduced to 150 nm after a single UST pass at 300 mPa. The krill oil formulation showed faster rates of particle size reduction and sample clarification than those of argan oil formulations (Figure 2). Mean particle size progressively decreased from 61 nm to 38 nm following two and three passes, respectively, and a minimum particle size of 24 nm was observed after eight passes.

### 2.4. Rate of Particle Size Reduction as a Function of Pressure

**Figure 3:**
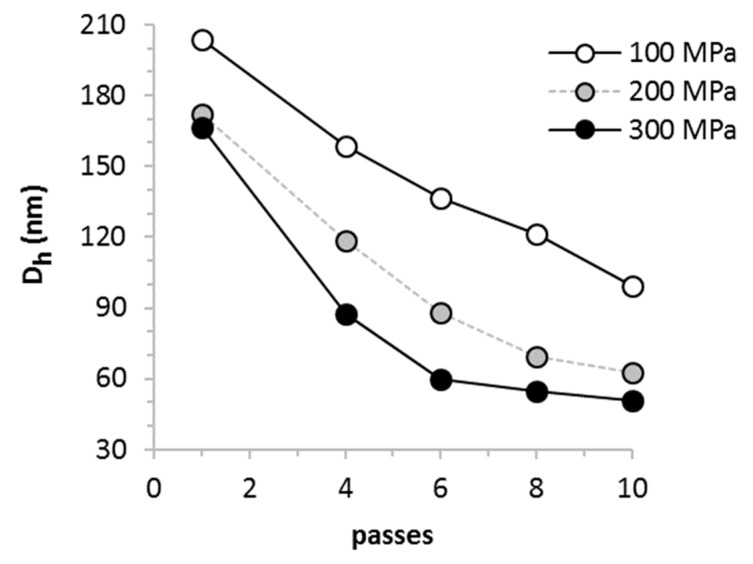
Rate of particle size reduction in a representative nanoemulsion as a function of pressure. Lipid particles smaller than 180 nm were observed following a single pass through the UST valve at 200 and 300 MPa. Minimal particle size was typically reached after 6–8 passes at 300 MPa. By comparison, minimal particle size was not reached at 100 or 200 MPa. Off-scale micron-sized oil droplets formed in the pre-UST samples were not plotted.

The polydisperse micron-sized particles formed in coarse emulsions were decreased in size to less than 180 nm following a single pass through the UST valve at 200 and 300 MPa pressure. Particle size progressively decreased with each additional pass with a characteristic inflection in the rate of particle size reduction typically observed after 4–6 passes at 300 MPa. This high rate of particle size reduction is of particular importance when processing food additives and nutraceuticals at the production scale. Minimal particle size was not reached within 10 passes at either 100 or 200 MPa (Figure 3).

### 2.5. Centrifugation Assay

The products were filtered, sterilized, and then centrifuged at 3000 RCF for 15 min prior to analysis. No phase separation (no floating layer) or precipitation of AsX was observed, suggesting that high efficiency of emulsion formation approaching 100% encapsulation was achieved by UST in these formulations. AsX concentration measured at 450 nm was identical to unfiltered, uncentrifuged samples. 

### 2.6. Stability of AsX/Argan Oil Nanoemulsions

**Figure 4:**
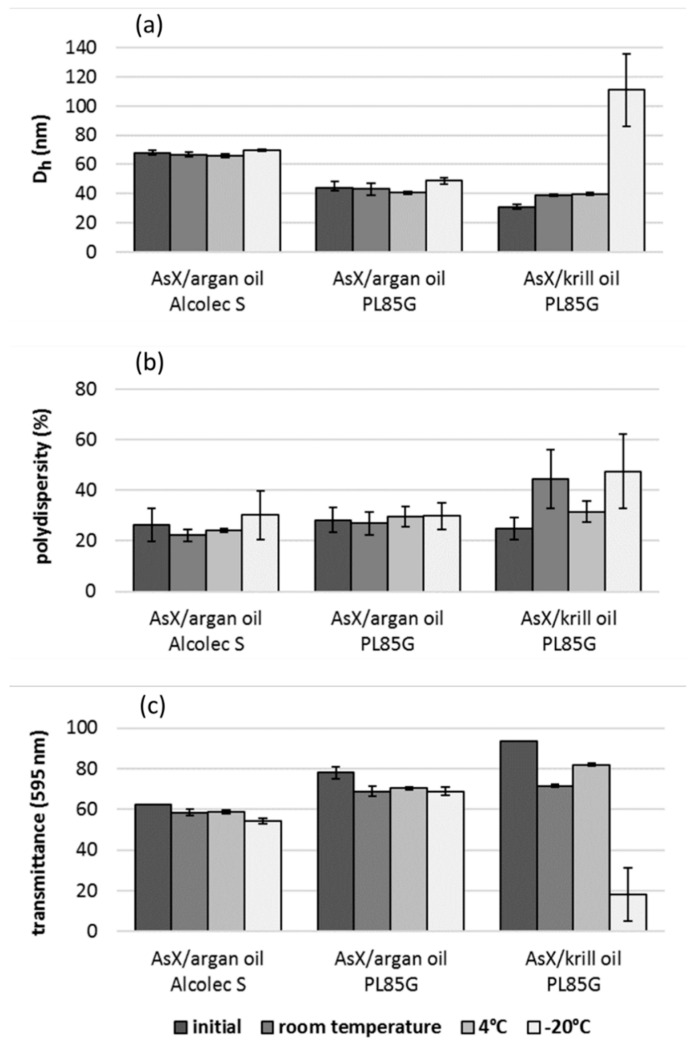
Stability of AsX/argan oil nanoemulsions stored for 90 days at room temperature and 120 days at 4 °C or −20 °C. AsX/krill oil nanoemulsions were stored for 60 days at room temperature and 90 days at 4 °C or −20 °C. Frozen samples were reanalyzed after one freeze–thaw cycle and centrifugation. No significant change in (**a**) particle diameter, (**b**) polydispersity, or (**c**) sample clarity (expressed as transmittance) was observed in AsX/argan oil formulations when compared to initial measurements taken within one hour of UST nanoemulsion preparation. Increased particle size concomitant with decreased clarity was observed in the krill oil formulation following a single freeze–thaw cycle.

AsX/argan oil nanoemulsions were shown to be stable for at least three months at room temperature using particle size, polydispersity, and sample clarity as criteria (Figure 4). Further, AsX/argan oil nanoemulsions were stable for at least four months when stored refrigerated. Both PL85G and Alcolec S argan oil formulations were stable following three months of storage at −20 °C with no significant change observed following a single freeze–thaw cycle.

### 2.7. Instability of AsX/Krill Oil Nanoemulsions

Stability studies of AsX/krill oil nanoemulsions showed an increase in the particle mean diameter concomitant with decreased sample clarity following storage for two months at room temperature or three months at 4 °C. Compared to initial measurements taken within an hour of nanoemulsion preparation, the mean particle size increased in a manner consistent with Ostwald ripening at both temperatures. Following −20 °C storage of AsX/krill oil nanoemulsion, the mean particle size was highly variable in replicate freeze–thaw experiments, typically doubling or tripling in size, strongly suggesting that coalescence had occurred. While AsX/krill oil nanoemulsions were increasingly turbid, no phase separation was observed after a single freeze–thaw cycle.

## 3. Discussion

Nanoemulsification by intense fluid shear by UST produces dispersions of extremely small oil droplets, which in turn, are stabilized by the adsorption of surfactant molecules on the particle surface. As smaller, more numerous lipid particles are formed, their collective surface area increases, and proportionately higher surfactant concentrations are required for stabilization, thus emphasizing the importance of optimized formulations. Otherwise, the nascent particles are susceptible to coalescence, and eventually phase separation.

The UST process uses pressure up to 400 MPa to accelerate fluids to velocities over 650 m/s. Combined with a self-throttling annular valve capable of a gap size significantly smaller than conventional fixed orifice homogenizers, higher shear rates consistently produced nanoemulsions of small mean particle size and high optical clarity. Particle sizes were reduced by two orders of magnitude following a single pass through the nanostructured valve in both argan oil and krill oil formulations. AsX/argan oil nanoemulsions were most stable with no measurable change following three months of storage at room temperature. The PL85G formulation produced a slightly smaller particle size than that of the Alcolec S formulation.

While the krill oil formulation yielded the smallest particle size of the three formulations investigated, these nanoemulsions were the least stable. Although krill oil would seem a logical carrier oil for the formation of AsX nanoemulsions, it contains a diverse class of lipids that can vary considerably according to the method of extraction [17]. Further studies are warranted to determine whether more or less refined krill oils might further improve nanoemulsion stability.

## 4. Materials and Methods

### 4.1. Materials

Argan oil was from Skin Actives Scientific (Gilbert, AZ, USA). Krill oil NKO from *Euphausia superba* was obtained from Jedwards International (Braintree, MA, USA). PL85G and Alcolec S lecithin derivatives were from American Lecithin Company (Oxford, CT, USA). PS20 was from Thermo Fisher Scientific (Waltham, MA, USA). PS80 was purchased from Lotion Crafters (Eastsound, WA, USA).

### 4.2. Conventional Rotor-Stator Homogenization of AsX Emulsions

Crude emulsions were prepared using an Omni TH115 rotor–stator homogenizer model with an Omni Tip homogenizer probe (Omni International, Kennesaw, GA, USA). AsX concentrations were 1.2 mg/g in argan oil and 0.4 mg/g in krill oil. The oil phase constituted 10–12% of the final formulations to which 10% glycerol was added for cryostability. Surfactants were 5% PS20 and either 4% PL85G or 4% Alcolec S. The total surfactant-to-oil ratio (SOR) was 0.7–0.9. Water content was 71%.

### 4.3. Ultra-Shear Technology (UST)

**Figure 5:**
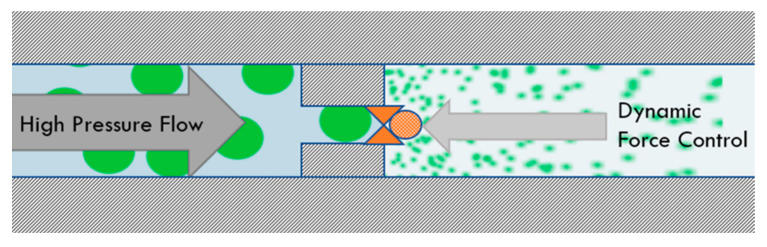
Schematic representation of the UST annular valve mechanism of action. The unprocessed product is forced through an annular gap between a ball and a seat of the homogenizer valve, while the ball is being actively pressed against the seat with a controlled force to maintain the minimal gap opening across a wide range of flow rates and material viscosity values. The valve gap is adjustable from 0–1000 nm.

Following homogenization, crude emulsions were immediately processed with the UST system (Pressure BioSciences, South Easton, MA, USA). For this, the sample is completely isolated in a disposable bladder, thus preventing direct contact of the sample with the mechanical features of the high-pressure pump and avoiding contamination from previously processed samples. For each passage, the bladder is filled with a sample using a low-pressure charge pump, then discharged through the UST valve at 300 MPa. The self-throttling annular valve gap is adjustable from 0–1000 nm (Figure 5).

**Figure 6:**
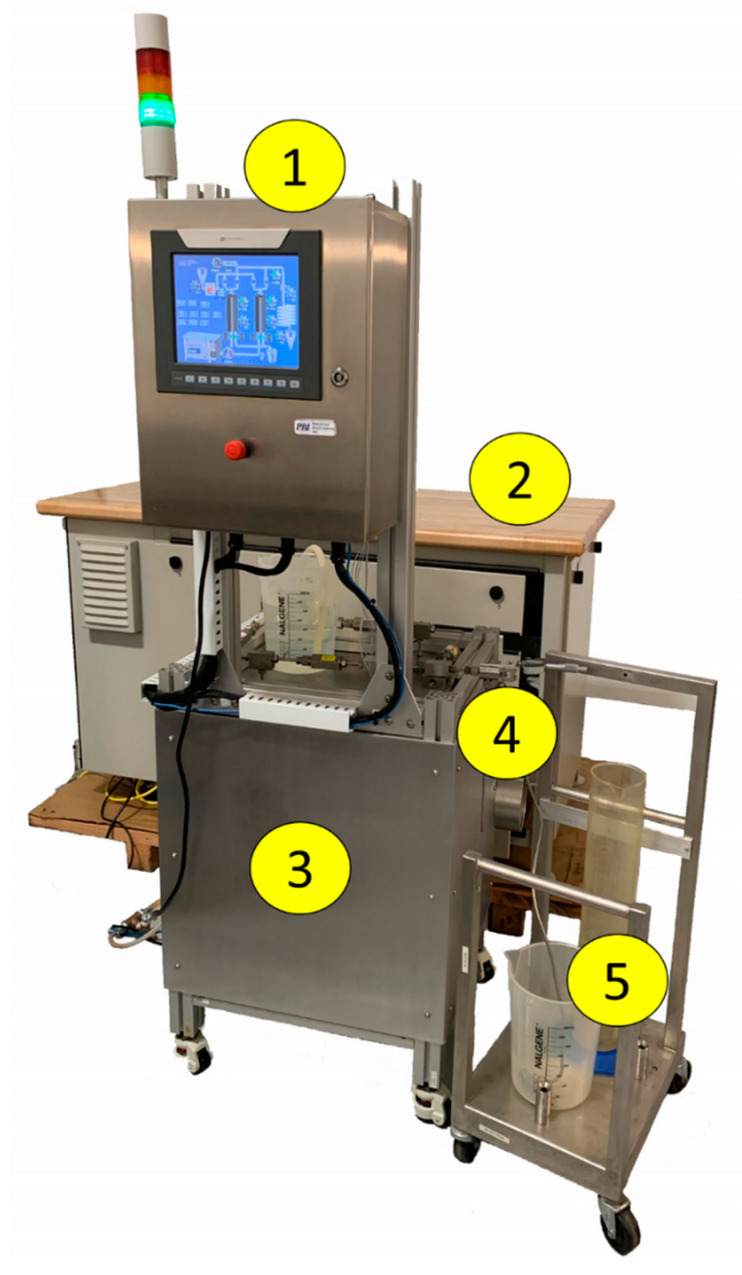
Industrial-scale UST homogenizer capable of continuous operation at pressure up to 400 MPa and flow rates up to 4 L/min. (1) Control system, (2) 40 horsepower, 400 MPa pump, (3) isolator enclosure, (4) UST valve, (5) output to filler.

UST uses a proprietary fluidic isolator to decouple the product from physical contact with the pump providing the high pressure. In the isolator, pressure is transmitted from the pump water to the product. This allows the use of existing non-food-specific high-performance pumps to achieve a high product flow rate at a lower cost. Furthermore, ultra high pressure operation of the self-throttling annular valve maximizes fluid velocity and minimizes gap distance to achieve shear rate up to 10^7^ s^−1^. This is significantly higher than is possible with conventional homogenizers. UST equipment currently in development is capable of continuous operation at pressure up to 400 MPa and flow rates up to 4 L/min (Figure 6).

### 4.4. Estimation of Oil Droplet Size in Crude Emulsions

Oil droplets formed in crude emulsions were too large and polydisperse to be reliably sized by DLS. For this, crude emulsions were analyzed on an etched glass hemacytometer (Hausser Scientific, Horsham, PA, USA). Mean oil droplet sizes were determined from micrographs using the ImageJ image analysis software [18,19].

### 4.5. Dynamic Light Scattering

Particle size and polydispersity were determined by dynamic light scattering (DLS) using a DynaPro NanoStar from Wyatt Technology (Goleta, CA, USA). Particle size was expressed as the mean hydrodynamic diameter (D_h_) of ten consecutive measurements.

### 4.6. UV/Vis Spectroscopy

UV/Vis spectroscopy was performed using a Biotek/Agilent 800 microplate reader (Winooski, VT, USA). Sample turbidity was determined from absorbance measurements at 595 nm and expressed as “clarity” by conversion to transmittance. Abs _595 nm_ correlated well with D_h_ determined by DLS (r^2^ = 0.9331) over the 20–180 nm size range. AsX encapsulation was quantified by absorbance 450 nm.

### 4.7. Filter Sterilization

Nanoemulsions of particle size <100 nm were filter sterilized using Sartolab RF50 vacuum filtration units with 0.22 micron polyether sulfone membrane from Sartorius (Göttingen, Germany). Products analyzed before and after filtration showed no significant difference in particle size, polydispersity, or AsX concentration.

### 4.8. Centrifugation Assay

Samples were centrifuged at 3000× *g* for 15 min prior to analysis.

### 4.9. Stability Studies

The storage stability of AsX/argan oil and AsX/krill oil nanoemulsions was studied in terms of particle size, polydispersity, sample clarity, and AsX concentration. Argan oil nanoemulsions were stored for 90 days at room temperature and 120 days at 4 °C or −20 °C. Krill oil nanoemulsion was stored for 60 days at room temperature and 90 days at 4 °C or −20 °C. Replicates of filter-sterilized nanoemulsions were stored frozen and reanalyzed after one freeze–thaw cycle following centrifugation.

## 5. Conclusions

Nanoemulsion technology holds significant potential for food, nutraceutical, and pharmaceutical applications. The ability to produce commercial nanoemulsion-based products would depend on large-scale production requiring minimal repeat passes for size refinement. It is evident that operating at higher pressure can reduce both the size of the nanoemulsion as well as the number of passes needed. Nevertheless, careful formulation is also important to achieve optimal dispersion formation. Nanoemulsions that show high stability in storage and freezing make commercial products more feasible. Efficient nanoemulsion formulation, production, and characterization methods will all grow in importance.

## 6. Patents

Patents issued and pending: ZL201620243518.2; ZL201621148183.2; US:15/562,146; EU:16773905.1; US CIP:62/907983; AU:2016243553; JP:2018-510061; 2018-526596; US-2019-0085982-A1; EU:3347630.

## Data Availability

Data is contained within the article.

## Sample Availability

Samples of the compounds are astaxanthin nanoemulsions, available from the authors.

## Abbreviations

AO, argan oil; AsX, astaxanthin; KO, krill oil; PL85G, Phospholipon 85G; PS20, polyoxyethylene sorbitan monolaurate; PS80, polyoxyethylene sorbitan monooleate, SOR, surfactant:oil ratio; UST, Ultra-Shear Technology.
